# Complete genome sequence of vaginal swab isolate *Enterococcus faecalis* UMB6935B, including two complete plasmids

**DOI:** 10.1128/mra.00368-25

**Published:** 2025-07-07

**Authors:** Olive Kolar, Helen Appleberry, Alan J. Wolfe, Alex Kula, Catherine Putonti

**Affiliations:** 1 Department of Biology, Loyola University Chicago 224010, Chicago, Illinois, USA; 2 Bioinformatics Program, Loyola University Chicago 2456, Chicago, Illinois, USA; 3 Department of Microbiology and Immunology, Stritch School of Medicine, Loyola University Chicago 12248, Chicago, Illinois, USA; Wellesley College, Wellesley, Massachusetts, USA

**Keywords:** *Enterococcus faecalis*, vaginal microbiome, overactive bladder symptoms

## Abstract

*Enterococcus faecalis* has been identified as a member of the vaginal community of healthy females, in addition to its association with vaginitis and urinary tract infections. Here we present the hybrid assembly of an *E. faecalis* strain, UMB6935B, isolated from a vaginal swab from a female with overactive bladder symptoms.

## ANNOUNCEMENT

Although a common resident of the gastrointestinal tract (1), *Enterococcus faecalis* within the urinary tract is often associated with symptoms or infections (2–5). While *E. faecalis* is frequently found in the vaginal community of healthy females (6), its presence has also been associated with miscarriages (7), peripartum infections (8, 9), and bacterial vaginosis (see review [10]). Prior research has suggested that the vaginal and urinary microbiota are interconnected (11, 12). We were thus prompted to further investigate *E. faecalis* isolated from the female urogenital tract.

*E. faecalis* UMB6935B was isolated from a vaginal swab from a female with overactive bladder symptoms as part of prior institutional review board-approved studies (LUC 207152 and 207777) (13). We obtained the strain from the Loyola Urinary Education and Research Collaborative collection and streaked it onto a brain heart infusion agar plate, which was incubated at 35°C with 5% CO_2_ for 24 hours prior to shipment to SeqCenter (Pittsburgh, PA, USA). There, DNA was extracted using the ZymoBIOMICS DNA Miniprep Kit, per the manufacturer’s protocol. One library was prepared using the DNA Prep kit (Illumina) and custom IDT 10 bp unique dual indices with a target insert size of 280 bp. This library was sequenced on an Illumina NovaSeq X Plus, and demultiplexing, quality control, and adapter trimming were performed with BCL Convert (v.4.2.4). With this same DNA, another library was prepared using the PCR-free Oxford Nanopore Technologies Ligation Sequencing Kit (SQK-NBD114.24) with the NEBNext Companion Module (E7180L) to manufacturer’s specifications. (No size selection or fragmentation was performed for the ONT library.) Sequencing was performed on an Oxford Nanopore MinION Mk1B sequencer or a GridION sequencer using R10.4.1 flow cells. Run design utilized the 400 bp sequencing mode with a minimum read length of 200 bp. Adaptive sampling was not enabled. Guppy (v.6.5.7) was used for super-accurate basecalling, demultiplexing, and adapter removal. SeqCenter delivered 3,270,734 2 × 151 bp paired-end Illumina read pairs and 278,839 long reads (*N*50 = 5,440 bp).

A hybrid assembly was performed using the web tool BV-BRC (v.3.36.16.5) (14) with the “auto” option. First, raw reads were trimmed via Trim Galore (v.0.6.5dev, https://github.com/FelixKrueger/TrimGalore) and normalized using BBNorm (v. 19 October 2017, https://jgi.doe.gov/data-and-tools/software-tools/bbtools/). The filtered reads were assembled using Unicycler (v.0.4.8) (15), and polishing was performed by two rounds of racon (v.1.4.20) (16) followed by two rounds of Pilon (v.1.23) (17). As part of the Unicycler tool, circular sequences were rotated to *dnaA*. The genome assembly was annotated upon submission to the National Center for Biotechnology Information (NCBI) using the NCBI Prokaryotic Genome Annotation Pipeline (v.6.9) (18). Sequences were also examined for antibiotic resistance genes using ResFinder (v.4.5.0) (19, 20). Replicon typing was performed by PlasmidFinder (v.2.1) (21). Unless otherwise specified, default parameters were used for all software tools.

The complete genome assembly consists of three contigs: one circular chromosome and two circular plasmid sequences. The short-read average coverage is 144.29×, and for long reads, it is 331.47×. Table 1 lists the attributes of the chromosome and plasmid sequences. The two plasmid replicon types are not unique to the vaginal community, as they have been identified in urinary as well as gut isolates (22), suggesting that *E. faecalis* UMB6935B may have been transferred to the vaginal community from one of these other two sites.

**TABLE 1:** Genome and plasmid sequence statistics

	Chromosome	pUMB6935B-1	pUMB6935B-2
Length (bp)	2,891,874	53,590	53,305
GC content (%)	37.5	34.7	33.4
No. of CDSs	2,727	61	59
No. of rRNAs	12	0	0
No. of tRNAs	62	0	0
Replicon (% identity)	n/a^*a*^	rep9c (97.35%)	rep9a (96.04%)
Antibiotic resistance genes	*lsa(A) tet(M*)	–^*b*^	*erm(B*)

^*a*^ n/a, not applicable.

^*b*^ –, no antibiotic resistance genes encoded by that plasmid.

## Data Availability

Both Illumina and Nanopore raw reads have been deposited at the National Center for Biotechnology Information and are available through the SRA database (accession nos. SRR32614272 and SRR32614273, respectively). The genome assembly has been deposited in GenBank under accession nos. CP185997 (chromosome), CP185998 (pUMB6935B-1), and CP185999 (pUMB6935B-2).
